# Sepsis Patients with First and Second-Hit Infections Show Different Outcomes Depending on the Causative Organism

**DOI:** 10.3389/fmicb.2016.00207

**Published:** 2016-02-26

**Authors:** Matt P. Morgan, Tamas Szakmany, Sarah G. Power, Patrick Olaniyi, Judith E. Hall, Kathy Rowan, Matthias Eberl

**Affiliations:** ^1^Division of Infection and Immunity, School of Medicine, Cardiff UniversityCardiff, UK; ^2^Directorate of Critical Care, Cardiff and Vale University Health BoardCardiff, UK; ^3^ACT Directorate, Cwm Taf University Health BoardLlantrisant, UK; ^4^Intensive Care National Audit & Research CentreLondon, UK; ^5^Institute of Life Science, College of Medicine, Swansea UniversitySwansea, UK; ^6^Systems Immunity Research Institute, Cardiff UniversityCardiff, UK

**Keywords:** sepsis, bacterial infections, intensive care, Gram-positive bacterial infections, Gram-negative bacterial infections

## Abstract

**Objective:** With improving rates of initial survival in severe sepsis, second-hit infections that occur following resolution of the primary insult carry an increasing burden of morbidity. However, despite the clinical relevance of these infections, no data are available on differential outcomes in patients with first and second-hit infections depending on the nature of the causative organism. This study aims to explore any differences in these subgroups.

**Design:** In a retrospective, observational cohort study, the United Kingdom Intensive Care National Audit & Research Centre (ICNARC) database was used to explore the outcomes of patient with first-hit infections leading to sepsis, and sepsis patients with second-hit infections grouped according to the Gram status of the causative organism.

**Setting:** General critical care units in England, Wales, and Northern Ireland participating in the ICNARC programme between 1 January, 2007 and 30 June, 2012.

**Patients:** Patient groups analyzed included 2119 patients with and 1319 patients without sepsis who developed an intensive care unit acquired infection in blood. Subgroups included patients with trauma, emergency neurosurgery, elective surgery, and cardiogenic shock.

**Measurements and main results:** Gram-negative organisms were associated with poorer outcomes in first-hit infections. The 90-day mortality of patients who developed a Gram-negative infection was 43.6% following elective surgery and 27.9% following trauma. This compared with a mortality of 25.6 and 20.6%, respectively, in Gram-positive infections. Unexpectedly, an inverse relationship between Gram status and mortality was observed in second-hit infections. Patients with an initial diagnosis of sepsis who developed secondary infections caused by Gram-negative organisms had a 90-day mortality of 40.4%, compared with 43.6% in Gram-positive infections.

**Conclusions:** Our study identifies a fundamental difference in patient outcomes between first-hit and second-hit bacterial infections, which may be due to genetic, microbiological, immunological, and environmental factors. This finding has direct implications for risk stratification and defines future research priorities.

## Introduction

Measured using any chosen metric, sepsis is a devastating condition for patients, their families, and society as a whole (Bryce et al., 2005; Newton et al., 2014). It accounts for 15–20% of all deaths in the developing world and kills over 1.5 million newborns and children every year (Bryce et al., 2005; Stevenson et al., 2014). As a medical condition, it is more deadly than stroke, killing a third of all patients with the severe form of the illness (Angus et al., 2001; Stevenson et al., 2014). It is responsible for a third of admissions to the intensive care unit (ICU) and costs the economy of the United States alone $17 billion annually (Angus et al., 2001; Schmid et al., 2002; Longo et al., 2007). For patients who do survive, many carry a substantial burden of continued physical and psychological ill health, with return to work rates below 65% (Bone et al., 1992; Schmid et al., 2002).

Large-scale surveillance studies have identified the most common organisms implicated in sepsis (Opal and Cohen, 1999; Vincent et al., 2009). Although fungal and viral infections contribute to many sepsis deaths, bacterial pathogens are the most frequent causative agents, with *Staphylococcus aureus* and *Streptococcus pneumoniae* representing the most relevant Gram-positive species, and *Escherichia coli, Klebsiella* spp., and *Pseudomonas aeruginosa* dominating the Gram-negative group (Angus et al., 2001; Vincent et al., 2009). The relative contribution by each of these different organism types is heavily influenced by local population characteristics, organism virulence, and health care structure variables.

The organism class responsible for the primary infection, has been shown to play a role in determining the mortality of patients with sepsis. In this study, these primary infections are termed “first-hit” infections. However, there are conflicting findings regarding the magnitude and the direction of the differences between Gram-positive and Gram-negative infections (Vincent et al., 2006; Labelle et al., 2012; Ani et al., 2015). The largest of these studies (Ani et al., 2015), with over 5 million patient records in the United States analyzed retrospectively, attributed a mortality of 30.4% to sepsis caused by Gram-positive organisms and 23.3% to Gram-negative organisms. However, the highest mortality in this cohort was 36.3% in patients infected with anaerobic Gram-negative microbes suggesting the importance of further stratification according to organism types instead of solely relying on Gram status.

With improving rates of initial survival in severe sepsis (Vincent et al., 1995; Gaieski et al., 2010; McPherson et al., 2013), nosocomial infections that occur following resolution of the initial insult carry an increasing burden of morbidity (Vincent et al., 1995; Agnese et al., 2002; Gaieski et al., 2010). In this study these infections are termed second-hit infections and include pathologies such as ventilator-associated pneumonia and intravascular line infections as well as reactivation of latent chronic viral infections such as cytomegalovirus (Vincent et al., 2009). However, despite the clinical relevance of these infections, there are no data available in the literature on differential outcomes from Gram-positive pathogens compared with Gram-negative species in patients with first and second-hit infections. We here attempted to address this knowledge gap, using both local data from a single hospital and data from a national audit database in the United Kingdom.

## Materials and methods

The design, management, and analysis of this observational cohort study were conducted according to the principles declared in The World Medical Association's Declaration of Helsinki. All data were analyzed anonymously, retrospectively, and did not impact upon the clinical care of any patients.

The definitions of sepsis and systemic inflammatory response syndrome (SIRS) were based on the 2012 Surviving Sepsis Guidelines in place at that time (Dellinger et al., 2013). The local data collection was approved by the South East Wales Research Ethics Committee (reference number 10WSE/421, June 2011) and registered with the UK Clinical Research Network (UKCRN; Cellular and biochemical investigations in sepsis, ID 11231).

The national data were screened from all admissions to NHS adult, general critical care units in England, Wales, and Northern Ireland participating in the Case Mix Programme of the Intensive Care National Audit & Research Centre (ICNARC) Data Specification between 1 January 2007 and 30 June 2012. An analysis plan was agreed *a priori* according to the following definitions:

**First-hit infection:** patients admitted with a non-infective diagnosis that subsequently developed an intensive care unit-acquired infection in blood.
**Second-hit infection:** patients admitted with severe sepsis as an initial diagnosis that subsequently developed an intensive care unit-acquired infection in blood.

All patients were categorized into those that developed Gram-positive or Gram-negative infection subtypes. Four specific patient subgroups were chosen before analysis as the first-hit cohort. These sub-groups were patients categorized as having trauma, emergency neurosurgery, elective surgery, and cardiogenic shock as their primary reason for intensive care admission. It has been shown that these patients can provide a plausible and accessible model of the development of severe sepsis (Cain et al., 2015).

As described above, patients in the second-hit cohort were admitted to the ICU with an initial diagnosis of severe sepsis, and then subsequently developed an ICU-acquired infection in blood. Thereafter, the same descriptive statistics and survival analyses were applied to patients with first-hit and second-hit infections. Acute hospital mortality was defined as the status at ultimate discharge from the acute hospital, excluding re-admissions within the same hospital stay.

The main organism causing the first-hit infection in blood was defined as the presence of an infection in any blood sample taken for microbiological culture 48 h or more following admission to the intensive care unit. Similarly, second-hit infection in blood was defined as the presence of infective bacteria in any blood sample taken for microbiological culture 48 h or more following admission to the intensive care unit in patients admitted with severe sepsis as initial diagnosis. If two organisms were isolated in both blood culture bottles, first organism priority was given according to the following ranking used by ICNARC: Methicillin resistant *S. aureus* (MRSA); *S. aureus* (not MRSA); vancomycin resistant *Enterococcus* spp. (VRE); *Enterococcus* spp. (not VRE); yeast (e.g., *Candida* spp.); *Pseudomonas* spp.; *Acinetobacter* spp.; *Enterobacter* spp.; *Klebsiella* spp.; *Serratia* spp.; *E. coli*; or other organisms entered using free text. The Gram classifications were then specified from the organism reported as the main organism causing first unit-acquired infection in blood.

The local dataset consisted of patients admitted with severe sepsis to the intensive care unit (ICU) at The Royal Glamorgan Hospital, Llantrisant, UK between 2010 and 2013 who were retrospectively analyzed for 90-day all-cause mortality according to the Gram status of the organism responsible for their initial sepsis diagnosis. Due to the narrow limits of this data collection restricted to electronically captured microbiological data and outcome data only, it was not possible to propensity match patients nor compare other cofounders such as age that may lead to excessive mortality in one arm of this study.

Cumulative survival curves as a function of time were generated using the Kaplan-Meier approach with censored results indicating patient discharge and compared using the log-rank test. Intergroup differences in baseline characteristics were compared using a two-way ANOVA, unmatched and corrected for multiple comparisons with a Sidak test using SPSS 20.0.

The funders had no role in study design, data collection, data analysis, data interpretation, or writing of the report. All authors had full access to all the data in the study and share final responsibility for the decision to submit for publication. The ICNARC data are available on request directly to icnarc@icnarc.org.

## Results

### Outcomes from first-hit infections according to local and national datasets

**Figure 1:**
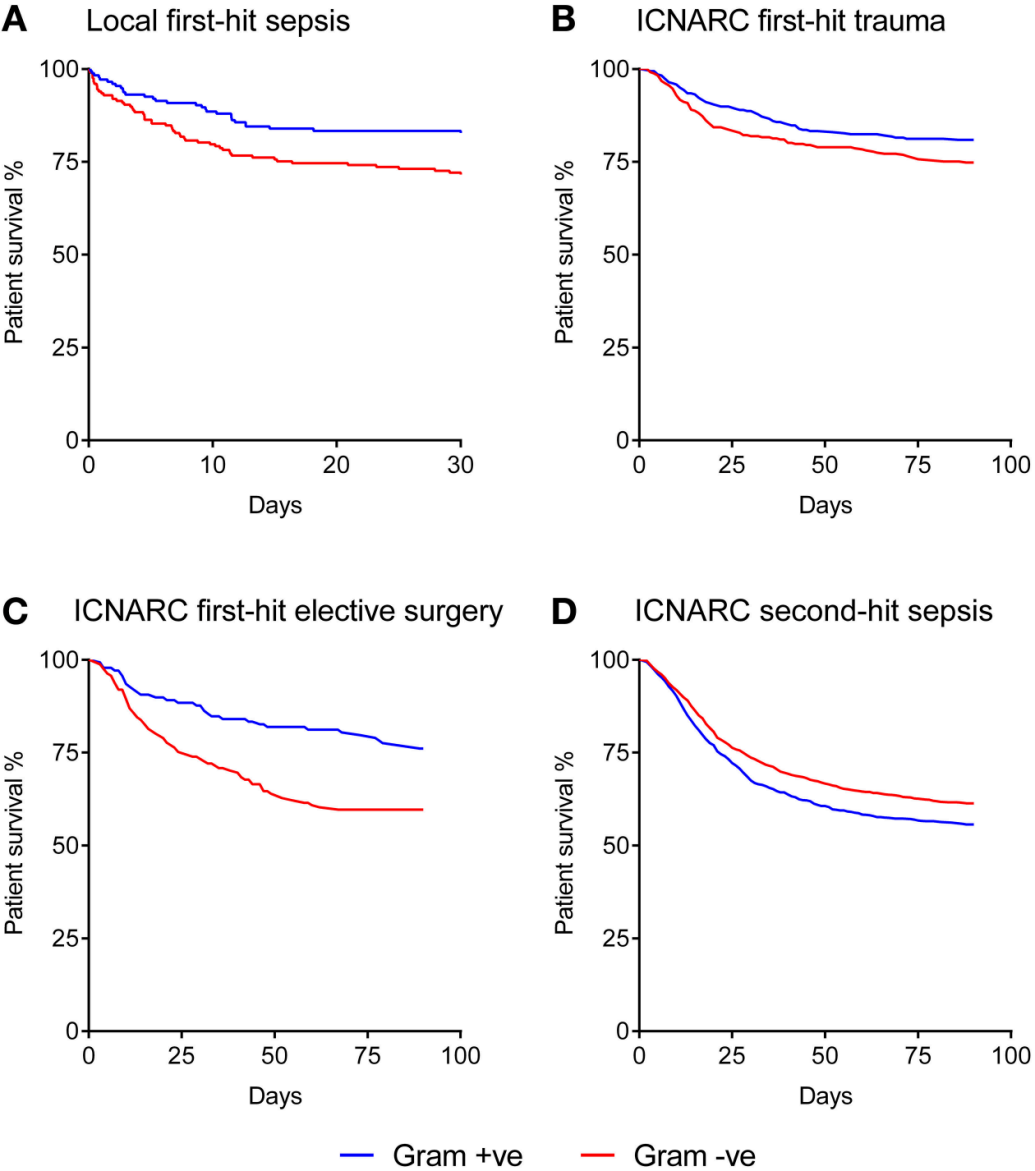
**Kaplan-Meier analysis of sepsis patient survival according to Gram status of the causative organism. (A)** Local dataset of first-hit sepsis patients (*n* = 371). **(B)** ICNARC dataset of first-hit trauma patients developing a unit-acquired infection (*n* = 703). **(C)** ICNARC dataset of first-hit elective surgery patients developing a unit-acquired infection (*n* = 616). **(D)** ICNARC dataset of second-hit sepsis patients subsequently developing a unit-acquired infection (*n* = 2131). All Gram differences are significant using the Mantel-Cox (Log-rank) test at *p* < 0.01.

The Kaplan-Meier curve shown in Figure 1A demonstrates that when a Gram-negative organism was identified as the prime cause of sepsis, patients had an excessive mortality rate of 29.1% compared with 21.3% for those where a Gram-positive organism was identified. This was equivalent to an odds ratio for death of 1.8 (1.18–2.73) in the Gram-negative subgroup.

In order to corroborate this relationship on a national scale, ICNARC's database of UK critical care units was used. ICNARC records do not include the causative organisms responsible for admissions to ICU with severe sepsis. The only recorded organism names are those responsible for “unit-acquired infections” occurring after 48 hrs or more following admission to ICU with alternative pathologies. We therefore identified groups of patients admitted to ICU without an infective etiology (trauma and elective surgery patients) to examine clinical outcome differences following acquisition of a unit-acquired infection that could act as a surrogate for first-hit infection causing severe sepsis.

The baseline characteristics of these groups of first-hit patients are shown in Table 1. While intergroup differences existed between trauma and elective surgery patients, as expected, the Gram-positive and Gram-negative groups within each cohort showed no significant differences in baseline parameters. Despite this similarity in morbidity, mortality rates showed striking differences between the two groups. Importantly, the mortality patterns in trauma (Figure 1B) and elective surgery patients (Figure 1C) matched that of the local dataset (Figure 1A). Mortality from Gram-negative infections in the trauma and elective groups was 27.9 and 43.6%, respectively, compared with 20.6 and 25.6% for Gram-positive infections. Overall, this translated to an odds ratio for death of 1.4 and 1.7, respectively, in trauma and elective surgery patients with Gram-negative infections. No significant differences were found in the mortality of patients with cardiogenic shock and those undergoing emergency neurosurgery although the numbers in these sub-groups were low (Supplementary Figure 1).

**Table 1:** **Baseline patient characteristics from the ICNARC dataset according to infecting organism type**.

	**First-hit (trauma)**		**First-hit (elective surgery)**		**Second-hit**	
	***Gram* +**	***Gram* −**	***Gram* +**	***Gram* −**	***Gram* +**	***Gram* −**
Number of admissions % [N]	49.8 [353]	49.4 [350]	46.4 [308]	53.2 [308]	47.3 [1009]	52.1 [1110]
Age mean (sd)	48.6 (20.0)	49.5 (19.8)	64.4 (14.6)	67.6 (12.8)	61.9 (14.9)	61.6 (15.4)
Gender % male	77.9	74.9	69.2	76.8	60.5	59.4
Caucasian %	88.6	90.6	95.6	96.9	94.0	92.4
Liver condition in PMH %	1.1	1.1	2.0	4.0	48.6	47.8
Renal condition in PMH %	0.0	1.7	0.0	2.0	1.7	1.6
Respiratory condition in PMH %	0.6	0.6	1.0	4.0	48.4	47.5
Cardiovascular condition in PMH %	0.3	0.3	2.0	4.0	0.9	0.8
In- hospital CPR %	0.8	4.0	2.0	3.0	3.1	2.3
Community CPR %	0.0	2.0	0.0	0.0	0.3	0.4
No CPR %	97.2	94.0	98.3	97.9	96.6	97.3
ICNARC mean (sd)	19.4 (6.7)	20.9 (7.2)	18.2 (8.3)	17.8 (7.3)	25.2 (8.1)	25.5 (8.1)
APACHE II mean (sd)	14.5 (6.2)	14.9 (6.7)	16.5 (5.8)	16.2 (4.7)	20.4 (6.3)	20.3 (6.5)
Acute hospital mortality %	20.6	27.9^**^	25.6	43.6^**^	46.3	40.4^**^

There were no significant differences in these baseline characteristics between organism types within respective groups to explain the mortality differences observed by Gram-status. The results were analyzed using a two-way ANOVA, unmatched and corrected for multiple comparisons with a Sidak test ^*^p < 0.05;

** p < 0.01;

^***^p < 0.001. PMH, past medical history; CPR, cardiopulmonary resuscitation; APACHE, Acute Physiology And Chronic Health Evaluation.

### National outcomes from second-hit infections

As ICNARC records unit-acquired organism names in different cohorts of patient groups, it was possible to examine the mortality in severe sepsis patients who develop a second-hit infection. The baseline characteristics of these patients had no statistical differences when using Gram status as a comparator (Table 1). However, compared with first-hit infections, an inverse relationship between Gram status and mortality was seen. Second-hit infections in sepsis patients had a mortality of 40.4% when a Gram-negative infection was responsible compared with 43.6% when Gram-positive organisms were recorded (Figure 1D). This resulted in an odds ratio for death of 0.8 following infection with Gram-negative pathogens in second-hit infections.

## Discussion

The present analysis accords with previous studies showing that first-hit infections caused by Gram-negative organisms result in a greater mortality in sepsis compared with Gram-positive pathogens (Ani et al., 2015). In striking contrast to this pattern in primary infections, our findings are the first to show that Gram-positive second-hit infections carry a higher risk of death compared to infections caused by Gram-negative pathogens. Of note, the national scale and standardized reporting of the corresponding data provide a significant advance in the analysis of differential outcomes in well-defined subgroups of patients developing first-hit or second-hit sepsis, that can now be addressed further in the clinic and experimentally.

Although infection-related organ dysfunction continues to be responsible for ~30% of ICU admissions, there is a surprising lack of comparative epidemiological data on the recent trends of infective organisms. The largest such dataset to-date, the EPIC II study, is almost 10 years old (Labelle et al., 2012). In that study, the investigators found a larger prevalence of Gram-negative infections and worse outcomes associated with certain organisms, and observed a significant relationship between time spent on the ICU and development of infections, particularly those caused by methicillin-resistant *S. aureus* (MRSA), *Acinetobacter*, and *Pseudomonas* species (Labelle et al., 2012). A small-scale study from mainland China recently confirmed this distribution of the infective organisms (Agnese et al., 2002). Our present findings demonstrate that the relative risk attributable to Gram-negative compared with Gram-positive mortality may be as high as 1.7 for first-hit infections.

The underlying causes for these mortality differences are likely to be multifactorial. Firstly, there may be logistical and procedural reasons as to why these patients have an excessive mortality. The increasing levels of multidrug-resistant Gram-negative organisms (Dellinger et al., 2013; Cain et al., 2015) may render patients with these causative organisms more likely to receive ineffective initial therapy (Micek et al., 2012; Zilberberg et al., 2014). However, recent data from the World Healthcare-Associated Infections Forum indicates that multidrug-resistant Gram-negative organisms only play a very small role in the UK with incidences below 5%, making this explanation less plausible (Leligdowicz et al., 2014).

Secondly, there may be unmeasured pathological differences due to the epidemiology of different organisms. In fact, after adjustments for organism class and type, the site of infection appears to play a key role in differential patient survival (Vincent et al., 2006; De Waele et al., 2014; Leligdowicz et al., 2014). With the knowledge that patterns of microbial classes differ between different infectious sources, simply basing a mortality prediction on an organism type may act as a surrogate for the likely source of infection. This may help explain some of the variation shown in the literature comparing organism class and species. The extent of variation shown in those studies exposes many of the difficulties inherent in retrospective analysis of a syndrome characterized by a number of individual disease entities across a hugely variable cohort of patients. In conjunction with widely varying microbial resistance patterns across different countries, the inconsistent use and timing of appropriate antibiotics makes comparing international results a difficult task and further highlights the need for better quality data.

Thirdly, the differences in outcome between Gram-negative and Gram-positive infections may represent a particular predisposition of different patients to develop distinct types of infections (Agnese et al., 2002; Escoll et al., 2003; Cavaillon and Adib-Conquy, 2006; Porta et al., 2009; Lynn, 2014). What has been observed in our study may simply be an excessive mortality due to genetic and environmental differences rather than the microorganisms directly. However, despite these possibilities, it is undeniable that the Gram status can be used as a strong signal to point toward an expected excessive mortality. This in itself is important and useful.

Finally, there are clear immunological differences that occur as a result of an organism's structural and biochemical characteristics. As a classical example, this may predominantly be due to the presence of a lipopolysaccharide (LPS)-containing cell wall in Gram-negative bacteria. LPS is recognized by a range of cell types and promotes inflammation as well as acts as potent inducer of the coagulation cascade (Mansur et al., 2014). In addition to the presence of LPS as a major discriminator between Gram-negative and Gram-positive bacteria, such mortality differences seen here may also be influenced by other pathogen-specific characteristics including the ability of most Gram-negative organisms to activate innate-like Vγ9/Vδ2 T cells and mucosal-associated invariant T (MAIT) cells (Davey et al., 2014; Grimaldi et al., 2014). Individual organism pathogenicity will also influence patient outcomes as much as the pharmacokinetics of the drugs used to target such microbes. Therefore, more virulent Gram-negative microbes may more rapidly replicate and have higher toxin loads (Ramachandran, 2014).

What is more intriguing than the relationship between Gram status and mortality from first-hit infection is the apparent inverse relationship between mortality and Gram status in sepsis patients who subsequently acquire a second-hit infection. Again, this is likely to be multifactorial. There is a wealth of immunological literature demonstrating profound reprogramming effects on both cellular and humoral immunity that severe sepsis leaves in its wake (Escoll et al., 2003; Cavaillon and Adib-Conquy, 2006; Trusheim et al., 2007; Porta et al., 2009; Hotchkiss et al., 2013; Davey et al., 2014; Lynn, 2014). These tolerising effects may render survivors of first-hit infections more resistant to subsequent Gram-negative sepsis. There may also be organizational aspects to these mortality differences including the use of antimicrobials with adverse side effect profiles in Gram-positive second-hit infections to cover the possibility of MRSA infection. Furthermore, there may be a survival bias to these data. For example, those patients who survive an initial Gram-negative infection may have an inherent resistance to Gram-negative infections. Therefore, these patients may be more likely to survive and subsequently develop second-hit infections, and the data might thus, be skewed toward a survival benefit of Gram-negative infection when these patients develop a second infection.

Several improvements could be made in future studies of this topic. Firstly, microbiological data on true first-hit sepsis patients were not available through ICNARC's dataset. Therefore, we defined surrogate first-hit infection subgroups including post-operative elective surgery and trauma patients. There is a large volume of research supporting the use of these groups of patients as a model for investigating first-hit infection (Cain et al., 2015). With the advent of new nation-wide systems of sepsis outcomes such as the recording through the work of the UK Sepsis Trust and the National Institute for Health and Care Excellence (NICE), causative organism data may be possible to analyse in the future. Indeed, new trial design may be a key component of improving research in this area (Stevenson et al., 2014). Studies should also aim to address the survival bias discussed above. By recording the initial infecting organism responsible for the first-hit sepsis, it should be possible to explore such relationships further.

Secondly, although the ICNARC dataset has considerable power due to its size and robust collection methods, it suffers from lack of granular detail and a relatively arbitrary collection priority of organisms. It would be important in future research to record when and from where individual organisms are isolated. The ranking of organisms allowing only a single species to be recorded may bias data collection in favor of Gram-positive infections that in turn may skew future analysis. The list of organisms was based on data from the European Centre for Disease Prevention and Control for the UK and has been shown to be representative in independent datasets (Heginbothom et al., 2013). Therefore, ICNARC outcome data have significant clinical relevance in everyday practice. In addition to these possible confounders, the disease severity scores in the present study were recorded at the time of ICU admission, rather than at the time of initial pathology (i.e., surgical procedure time point), and may thus have diverged by the time of subsequent ICU admission. Unfortunately, the ICNARC dataset is not able to compensate for these factors. However, despite these methodical issues, they remain constant in all groups studied and as such cannot account for the reversal of mortality in first-hit compared with second-hit infections. This is a clear signal being sent although the intricacies of this detail will need to be addressed with a different future methodology.

## Conclusion

Overall, our study demonstrates that Gram-negative infections are associated with a greatly elevated mortality in first-hit sepsis patients whilst these differences are reversed in second-hit infections. These findings will allow clinicians to better plan and deliver care to the patients most at risk from severe sepsis by targeting resources more effectively. It may also form a platform to explore the immune reprogramming effects of sepsis *ex vivo* by comparing subsequent responses from patients with differing initial infection types.

## Author contributions

MM, KR, and ME designed the study. TS and PO abstracted the local data. SP and KR abstracted the national data from the Case Mix Programme. MM, SP, and KR directed and conducted the data analysis. MM, TS, and ME wrote the paper. JH provided expert advice and revised the draft. All authors read and approved the final version.

## Funding

Dr. TS reports grants from National Institute of Social and Health Care Research, Welsh Assembly Government, UK, during the conduct of the study. Dr. ME reports grants from SARTRE/SEWAHSP Health Technology Challenge and the MRC Confidence in Concept scheme, during the conduct of the study.

### Conflict of interest statement

The authors declare that the research was conducted in the absence of any commercial or financial relationships that could be construed as a potential conflict of interest.

## Supplementary material

Supplementary Figure 1 **Kaplan-Meier analysis of sepsis patient survival according to Gram status of the causative organism. (A)** ICNARC dataset of first-hit emergency neurosurgical patients developing a unit-acquired infection (*n* = 104). **(B)** ICNARC dataset of first-hit cardiogenic shock patients developing a unit-acquired infection (*n* = 56). None of the Gram differences are significant using the Mantel-Cox (Log-rank) test at *p* < 0.05.
