# Supplementary figures and images for: Sepsis Patients with First and Second-Hit Infections Show Different Outcomes Depending on the Causative Organism
Source: Front Microbiol. 2016 Feb 26;7:207. doi: 10.3389/fmicb.2016.00207 (PMC4767904; doi:10.3389/fmicb.2016.00207)

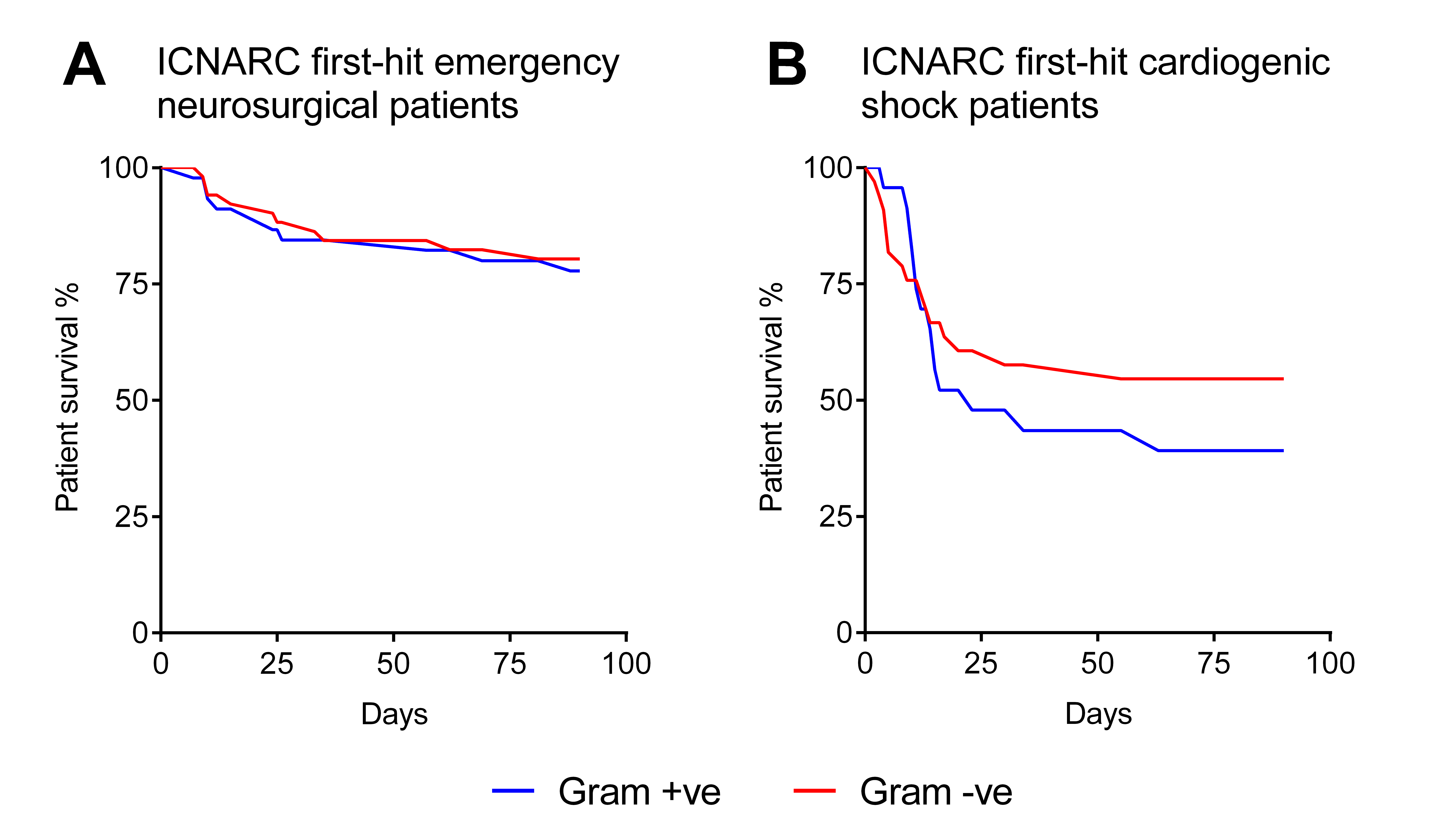

Supplement: Supplementary Figure 1 — Kaplan-Meier analysis of sepsis patient survival according to Gram status of the causative organism. (A) ICNARC dataset of first-hit emergency neurosurgical patients developing a unit-acquired infection (n = 104). (B) ICNARC dataset of first-hit cardiogenic shock patients developing a unit-acquired infection (n = 56). None of the Gram differences are significant using the Mantel-Cox (Log-rank) test at p < 0.05. [file Image1.tif]
